# Peripheral Immune Cell Ratios and Clinical Outcomes in Seropositive Autoimmune Encephalitis: A Study by the Australian Autoimmune Encephalitis Consortium

**DOI:** 10.3389/fimmu.2020.597858

**Published:** 2021-01-14

**Authors:** James Broadley, Robb Wesselingh, Udaya Seneviratne, Chris Kyndt, Paul Beech, Katherine Buzzard, Cassie Nesbitt, Wendyl D’Souza, Amy Brodtmann, Tomas Kalincik, Helmut Butzkueven, Terence J. O’Brien, Mastura Monif, Australian Autoimmune Encephalitis Consortium

**Affiliations:** ^1^ Department of Neuroscience, Monash University, Melbourne, VIC, Australia; ^2^ Department of Neurology, Alfred Health, Melbourne, VIC, Australia; ^3^ Department of Neuroscience, Monash Health, Melbourne, VIC, Australia; ^4^ Department of Medicine, St Vincent’s Hospital, University of Melbourne, Melbourne, VIC, Australia; ^5^ Department of Neurosciences, Eastern Health, Melbourne, VIC, Australia; ^6^ Department of Neurology, Melbourne Health, Melbourne, VIC, Australia; ^7^ Department of Radiology, Alfred Health, Melbourne, VIC, Australia; ^8^ Department of Radiology, Monash Health, Melbourne, VIC, Australia; ^9^ Department of Neuroscience, Barwon Health, Geelong, VIC, Australia; ^10^ Department of Medicine, The University of Melbourne, Melbourne, VIC, Australia; ^11^ Clinical Outcomes Research, The University of Melbourne, Melbourne, VIC, Australia; ^12^ Department of Physiology, The University of Melbourne, Melbourne, VIC, Australia

**Keywords:** autoimmune encephalitis, neutrophil-to-lymphocyte ratio, monocyte-to-lymphocyte ratio, prognosis, biomarker

## Abstract

**Objective:**

To examine the utility of the peripheral blood neutrophil-to-lymphocyte ratio (NLR) and monocyte-to-lymphocyte ratio (MLR) as biomarkers of prognosis in seropositive autoimmune encephalitis (AE).

**Methods:**

In this multicenter study, we retrospectively analyzed 57 cases of seropositive AE with hospital admissions between January 2008 and June 2019. The initial full blood examination was used to determine each patients’ NLR and MLR. The modified Rankin Scale (mRS) was utilized to assess the patients’ follow-up disability at 12 months and then at final follow-up. Primary outcomes were mortality and mRS, while secondary outcomes were failure of first line treatment, ICU admission, and clinical relapse. Univariate and multivariable regression analysis was performed.

**Results:**

During initial hospital admission 44.7% of patients had unsuccessful first line treatment. After a median follow-up of 700 days, 82.7% had good functional outcome (mRS ≤2) while five patients had died. On multivariable analysis, high NLR was associated with higher odds of first line treatment failure (OR 1.32, 95% CI 1.03–1.69, p = 0.029). Increased MLR was not associated with any short or long-term outcome.

**Conclusions:**

NLR on initial hospital admission blood tests may be provide important prognostic information for cases of seropositive AE. This study demonstrates the potential use of NLR as a prognostic marker in the clinical evaluation of patients with seropositive AE.

## Introduction

The interaction between the immune cells of the periphery and the “immune-privileged” CNS in autoimmune disorders has drawn significant attention. Many of the immunopathological models of CNS autoimmunity are based on multiple sclerosis (MS) and experimental autoimmune encephalomyelitis (EAE) in animals. In the last decade, the increased recognition of autoimmune encephalitis (AE) as a clinical entity has resulted in subsequent pathological studies in humans and animal models suggesting potential roles for innate and adaptive immune cell subsets, as well as their associated cytokines and chemokines, in disease pathogenesis (1).

Routine full blood examination is easily performed and provides a detailed snapshot of the circulating innate and adaptive immune cells. The neutrophil-to-lymphocyte ratio (NLR) is an increasingly recognized biomarker of systemic inflammation across a broad variety of different disease states including ulcerative colitis, rheumatoid arthritis, and systemic lupus erythematosus (2–6). In MS, the NLR has been demonstrated to correlate with disease progression and worse disability outcomes (7–9). Recent studies in AE have found that a high NLR is significantly correlated with long-term functional disability as measured by the mRS and reduced response to first line immunotherapy (10, 11). The monocyte-to-lymphocyte (MLR) has recently been associated with disability scores in MS (9). However, no published studies have examined the MLR as a prognostic biomarker in AE.

Here we performed a large multicenter retrospective study to examine for the presence of correlation between acute peripheral blood immune cell ratios, and morbidity and mortality in seropositive AE. We hypothesized that increased NLR and MLR would be associated with worse outcomes.

## Methods

### Patient Cohort

Ethics for this multicenter study was obtained through the Melbourne Health Human Research Ethics Committee. Patients were included from six secondary and tertiary referral centers in metropolitan hospitals in Victoria, Australia: Melbourne Health, Alfred Health, Monash Health, Eastern Health, Barwon Health, St Vincent’s Health. De-identified study data was collected and managed using REDCap (Research Electronic Data Capture) (12, 13). Potential cases were identified retrospectively through a medical records search of International Classification of Disease (ICD-10) codes for a principal or other diagnosis of AE with hospital admissions between January 2008 and June 2019. We also identified potential cases by performing a search of positive antibody results in the one site that had their own laboratory for antibody testing, which is a state-wide reference facility for receiving and processing samples for autoantibodies. Cases of AE were confirmed by reviewing each clinical record for diagnostic criteria, as established in recent guidelines (14). Only cases with a positive serum and/or cerebrospinal fluid (CSF) antibody test for neuronal cell surface antibodies were included in the study. Antibodies to NMDAR, AMPAR, GABAb, and IgLON5 were detected using commercial cell-based assays, while antibodies to LGI1 and CASPR2 were detected *via* direct immunofluorescence. Cases with autoantibodies to the voltage-gated potassium channel (VGKC) complex with negative or no specific testing for anti-LGI1 or anti-CASPR2 antibodies were excluded from the study.

We obtained data on demographics, antibody profile, clinical features, paraclinical findings (peripheral immune cell counts, CSF, and neuroimaging findings), follow-up MRI findings, treatment types and duration, and data on hospital stay (duration of admission, need for ICU admission). For this study, an abnormal MRI was defined as having findings that were in keeping with neuroinflammation (15). Patients had to be admitted to ICU for at least 48 h to be classified as having an ICU admission. This ensured patients with an ICU stay had critical illness, rather than those only needing a brief period of additional support or observation.

### Peripheral Immune Cell Counts Analysis

We recorded the total white blood cell count and immune cell subset counts from the first full blood analysis within 24 h of admission (10). We excluded any patient who had confounding illness that with potential impact on white blood cell counts such as systemic infection. We also excluded patients who had any form of immunotherapy prior to sampling. We noted patients who had generalized convulsive seizure activity within 12 h before sampling but did not exclude them from the cohort.

### Treatments

Immunotherapies were classified as first line (corticosteroids, intravenous immunoglobulin, plasma exchange) or second line (e.g. rituximab, cyclophosphamide) as previously described (16). Steroid sparing agents included mycophenolate mofetil, azathioprine, and methotrexate. We defined treatment failure as lack of improvement in the mRS by 1 or more point in the first 4 weeks of treatment initiation. This was based on conventions adopted in major publications in this field (16, 17).

### Clinical Outcomes

The primary endpoints were functional outcome [as assessed by the mRS (18)] at 12 months, mRS at longest follow-up, and mortality (both in hospital and at any point after symptom onset). If the mRS was not detailed at follow-up, it was determined retrospectively based on the case notes. A good outcome was described as an mRS of 2 or less (functional independence), consistent with most prior publications (16, 19–22). Secondary outcomes were failure of first line treatment, ICU admission during initial presentation, and clinical relapse. Follow-up was determined from the time of diagnosis. We documented the presence of relapse, which was defined as the re-occurrence of seizures, worsening reported cognitive deficits or worsening mRS (of 1 point or more) after a 1-month period of stabilization [adapted from reference (16)].

### Statistical Analysis

Baseline demographic, clinical, and paraclinical data were compared using summary statistics. A composite group containing all non-NMDAR patients was generated to compare against the NMDAR group. Outcomes were compared between these groups using Fischer’s exact test. Mann Whitney U test was used to compare the length of follow-up between the groups. The MLR values of the cohort were heavily right-skewed, so we performed a Box-Cox transformation using a λ of 0.5 to normalize the data (Shapiro-Wilk test 0.163). This variable was named “MLR transformed.”

Univariate logistic regression analyses were performed to determine correlations between selected covariates (including NLR and MLR) and the time-independent outcomes (mRS at 12 months, ICU admission, first line treatment failure). Univariate Cox regression analyses were performed to similarly examine the time-dependent outcomes (mortality, final mRS, relapse). For the purpose of the Cox regression analyses, patients that died during their initial inpatient admission were defined as having follow-up of 0 days. Clinically or statistically relevant variables from univariate analyses were used in the relevant multivariable logistic and Cox regression models, depending on the outcome of interest. Acknowledging the differences between the NMDAR group and all others (**Tables 1** and **2**), NMDAR serostatus was added as a covariate in the multivariable regressions (with the exception of the mortality and relapse regressions, as no patient with anti-NMDAR antibody-mediated encephalitis died and the two that relapsed did not have adequate blood samples). Patients with inadequate full blood examination were excluded from the regression analyses. Patients lost to follow-up were also excluded from analyses of post-discharge outcomes (mRS, mortality, and relapse). The cut-off for significance was a p-value less than 0.05. SPSS (IBM Corporation, version 26) was used for all statistical analyses.

**Table 1:** Demographic, clinical, paraclinical, and treatment data for each antibody subtype.

	All cases	NMDAR	Non-NMDAR	LGI1	CASPR2	AMPAR	GABAbR	IgLON5
**N**	57	29	28	19	4	3	1	1
**Female (%)**	57.9	79.3	35.7	42.1	0	33.3	100	0
**Age in years (median, range)**	55 (16–85)	28 (16–85)	66 (19–81)	67 (50–81)	67.5 (55–72)	59 (19–68)	68	64
**Tumor (%)**	0 (0–2)	27.6	10.7 (p = 0.179)	0	0	66.7	100	0
**Tumor type (n)**		Ovarian teratoma (8)				Ewing’s sarcoma (1), medullary thyroid (1)	Lung (1)	
**Median titer** **(pM, Serum)**				489	333.5			
**Abnormal CSF** **(n, %)**	42 (79.2)	23 (82.1)	19 (76)	10 (62.5)	4 (100)	3 (100)	1 (100)	1 (100)
**Abnormal MRI** **(n, %)**	17 (34.7)	4 (16.7)	13 (52)	10 (58.8)	1 (33.3)	2 (66.7)	0	0
**Treated (n, %)**	56 (98.2)	28 (96.6)	28 (100)	19 (100)	4 (100)	3 (100)	1 (100)	1 (100)

AMPAR, α-Amino-3-hydroxy-5-Methyl-4-isoxazolepropionic Acid Receptor; Caspr2, Contactin-Associated Protein-like 2; CSF, Cerebrospinal Fluid; GABA_B_R, γ-Aminobutyric Acid Receptor B; LGI1, Leucine-rich Glioma-Inactivated protein 1; MRI, Magnetic Resonance Imaging; mRS, modified Rankin Scale; NMDAR, N-Methyl-_D_-Aspartate Receptor.

**Table 2:** Median and range of immune cell subsets and ratios for each antibody subtype.

	All cases	NMDAR	Non-NMDAR	LGI1	CASPR2	AMPAR	GABAbR
**Total WCC, ×10^9^ cells/L** **(median, IQR)**	8.65 (3.9)	8.6 (2.5)	8.7 (4.7) (p = 0.750)	8.1 (4.6)	9.5	5.1	11.3
**Neutrophils, ×10^9^ cells/L** **(median, IQR)**	5.94 (3.3)	6.1 (2.9)	5.5 (3.55) (p = 0.239)	5.4 (3.8)	6	3.5	9.4
**Monocytes, ×10^9^ cells/L** **(median, IQR)**	0.7 (0.4)	0.6 (0.4)	0.7 (0.4) (p = 0.981)	0.7 (0.4)	1	0.2	0.4
**Lymphocytes, ×10^9^ cells/L** **(median, IQR)**	1.4 (1.1)	1.5 (0.76)	1.4 (1.3) (p = 0.786)	1.1 (0.9)	2.3	0.8	1.49
**NLR** **(median, IQR)**	4.27 (4.00)	4.51 (4.25)	4.00 (3.71) (p = 0.300)	4.20 (3.47)	2.50	5.00	6.28
**MLR** **(median, IQR)**	0.46 (0.38)	0.47 (0.41)	0.45 (0.35) (p = 0.795)	0.49 (0.32)	0.42	0.25	0.27

AMPAR, α-Amino-3-hydroxy-5-Methyl-4-isoxazolepropionic Acid Receptor; Caspr2, Contactin-Associated Protein-like 2; GABA_B_R, γ-Aminobutyric Acid Receptor B; IQR, interquartile range; LGI1, Leucine-rich Glioma-Inactivated protein 1; MLR, Monocyte-to-Lymphocyte Ratio; mRS, modified Rankin Scale; NLR, Neutrophil-to-Lymphocyte Ratio; NMDAR, N-Methyl-_D_-Aspartate Receptor; WCC, White Cell Count.

## Results

There were 57 patients that met our inclusion criteria. Cases with antibodies to the NMDAR were the largest overall constituent with 29 subjects. There were no patients with both LGI1 and CASPR2 antibodies, and there were only single cases with either γ-Aminobutyric Acid Receptor B (GABAb) or IgLON5 antibodies. We replicated prior data that adult anti-NMDAR antibody-mediated encephalitis is typically a disease of young females. Baseline demographic, clinical, paraclinical, and treatment data is shown in **Table 1**. An underlying neoplasm was found in 27.6% of NMDAR cases, all of which were ovarian teratomas. By comparison, patients withLGI1 or CASPR2 antibodies had no tumor diagnoses. Most people were treated with first line immunotherapy in the form of corticosteroids, intravenous immunoglobulin (IVIg), plasma exchange, or a combination thereof. There was only one patient that was untreated. According to the case notes, this patient was diagnosed in clinic with anti-NMDAR AE after the antibody result returned sometime after her discharge. There was a considerable delay to her follow-up, and by the time she was seen her symptoms had significantly improved and the treating team elected not to pursue immunotherapy. Twenty-five patients (43.9%) went on to have second line immunotherapy, while 15.7% of those who were followed-up received steroid-sparing agents during the follow-up period.

**Figure 1:**
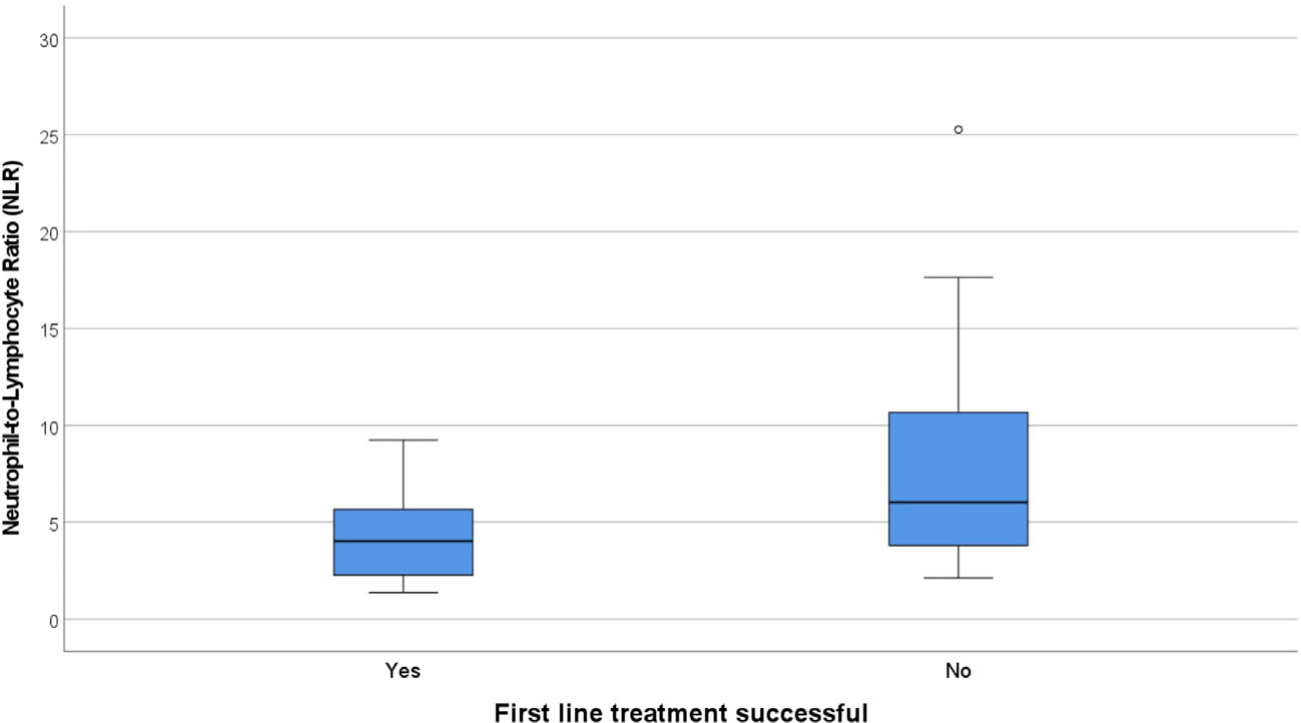
Box and whisker plots showing the median NLR in responders to first line treatment (4.02) vs non-responders (6.03). NLR, Neutrophil-to-Lymphocyte Ratio.

There were 12 cases that did not have an adequate full blood examination for our analysis, either because the result was unavailable or they had immunotherapy treatment prior (eight NMDAR, two LGI1, one CASPR2, one IgLON5). No patients had concomitant illness at the time of sampling, and therefore none were excluded on these grounds. The immune cell counts were similar between the NMDAR and Non-NMDAR patients (**Table 2**). NLR was higher in treatment non-responders than in responders (median 6.03 *vs* 4.02, **Figure 1**).

Five patients were lost to follow-up after hospital discharge (four NMDAR, one LGI1). The median duration of follow-up for the remaining 52 patients was 700 days (1.92 years), which was not different between the subgroups. Morbidity, follow-up, and outcome data were notable for significantly higher rates of ICU admission and mechanical ventilation amongst anti-NMDAR antibody-mediated encephalitis patients compared to those in the Non-NMDAR group (**Table 3**). The most common indications for ICU admission were behavioral disturbance or reduced conscious state. There was only one patient with anti-NMDAR antibody-mediated encephalitis with status epilepticus, and long-term outcomes in patients in this AE subset were generally good with no deaths. Poor functional outcome (as defined by mRS ≥3) at final follow-up occurred in a single patient with NMDAR AE. At the age of 85, this subject was the oldest in the NMDAR cohort. The non-NMDAR group had a lower rate of good functional outcome and higher mortality rate. At last follow-up, the single patient with GABAb encephalitis was deteriorating rapidly and not responding to immunotherapy with corticosteroids, intravenous immunoglobulin, and rituximab. Her expected prognosis was very poor, however she was lost to follow-up at 12 months. Rates of relapse and first line treatment response were no different between the groups. No patient with a tumor relapsed.

**Table 3:** Morbidity, follow-up, and outcome data for each antibody subtype.

	All cases	NMDAR	Non-NMDAR	LGI1	CASPR2	AMPAR	GABAbR	IgLON5
**ICU admission** **(n, %)**	19 (35.2)	**15 (53.6) (p = 0.004)**	4 (15.4)	2 (11.1)	1 (25)	0	1 (100)	
**Mechanical ventilation** **(n, %)**	14 (25.9)	**11 (39.3) (p = 0.030)**	3 (11.5)	1 (5.6)	1 (25)	0	1 (100)	
**Response to first line treatment (n, %)**	26 (55.3)	12 (50)	14 (60.9) (p = 0.561)	11 (68.8)	2 (50)	1 (50)	0	
**Mortality (n, %)**	5 (8.8)	0	**5 (17.9) (p = 0.023)**	2 (10.5)	0	2 (66.7)	0	1 (100)
**Cause of death (n)**				Pneumonia (1), cardiac arrest (1)		Upper GI bleed (1), cancer (1)		Type 2 respiratory failure (1)
**12-month mRS <3 (n, %)**	26 (76.5)	13 (81.3)	13 (72.2) (p = 0.693)	8 (72.7)	3 (100)	1 (50)	0	1 (100)
**Final mRS <3 (n, %)**	43 (82.7)	24 (96)	**19 (70.4) (p = 0.025)**	14 (77.8)	4 (100)	1 (33.3)	0	0
**Relapse (n, %)**	8 (15.7)	2 (8)	3 (23.1) (p = 0.248)	5 (29.4)	1 (25)	0	0	0
**Follow-up, days** **(median, IQR)**	700 (880)	865 (993)	585 (660) (p = 0.155)	510 (826)	810 (784)	455	365	1,035

Data is missing for the IgLON5 subset as we did not have access to that information. Statistics are performed comparing the NMDAR group against a set containing all other cases (non-NMDAR). Significant values (p < 0.05) are highlighted in bold. AMPAR, α-Amino-3-hydroxy-5-Methyl-4-isoxazolepropionic Acid Receptor; Caspr2, Contactin-Associated Protein-like 2; GABA_B_R, γ-Aminobutyric Acid Receptor B; GI, Gastrointestinal; ICU, Intensive Care Unit; IQR, interquartile range; LGI1, Leucine-rich Glioma-Inactivated protein 1; mRS, modified Rankin Scale; NMDAR, N-Methyl-_D_-Aspartate Receptor; STEMI, ST-Elevation Myocardial Infarction.

On univariate analysis, younger age, female sex, and failure of first line treatment were associated with ICU admission (**Table 4**). Interestingly, treatment delay did not have any significant relationship with an outcome. Increasing NLR was associated with greater odds of first line treatment failure (OR 1.27, 95% CI 1.01–1.60, p = 0.043) and mortality (OR 1.12, 95% CI 1.01–1.25, p = 0.041). Males had increased odds of relapse (HR 8.76, 95% CI 1.05–73.0, p = 0.045).

**Table 4:** Univariable logistic regression analysis for the whole cohort.

	Mortality	mRS ≤2 at 12-months	mRS ≤2 at final	ICU admission	Failure of first line treatment	Relapse
**Age (years)**	1.03 [0.98–1.08](p = 0.199)	**0.95 [0.91–0.99](p = 0.047)**	1.01 [0.99–1.02](p = 0.525)	**0.96 [0.94–0.99](p = 0.014)**	0.99 [0.96–1.01](p = 0.305)	1.03 [0.99–1.08](p = 0.111)
**Sex (Ref: female)**	6.07 [0.67–54.8](p = 0.108)	1.22 [0.24–6.23](p = 0.809)	1.09 [0.58–2.06](p = 0.784)	**0.10 [0.02–0.50](p = 0.005)**	0.58 [0.18–1.92](p = 0.375)	**8.76 [1.05–73.0](p = 0.045)**
**Tumor**	1.09 [0.12–9.80](p = 0.936)	1.21 [0.03–1.41](p = 0.110)	1.01 [0.47–2.20](p = 0.972)	2.77 [0.72–10.7](p = 0.140)	1.29 [0.28–5.94](p = 0.740)	0.04 [0.00–163](p = 0.441)
**Treatment delay (days)**	0.99 [0.97–1.01](p = 0.434)	1.04 [0.99–1.09](p = 0.135)	1.00 [0.99–1.00](p = 0.963)	1.00 [0.99–1.00](p = 0.245)	1.00 [0.99–1.01](p = 0.587)	1.00 [1.00–1.01](p = 0.372)
**Failure of first line treatment**	68.3 [0.01–740971](p = 0.373)	0.217 [0.03–1.36](p = 0.102)	0.55 [0.28–1.10](p = 0.091)	**7.88 [1.95–31.7](p = 0.004)**	–	0.20 [0.02–1.85](p = 0.157)
**Total WCC (×10^9^ cells/L)**	1.02 [0.86–1.26](p = 0.838)	1.07 [0.87–1.32](p = 0.510)	0.98 [0.90–1.06](p = 0.540)	1.06 [0.91–1.23](p = 0.482)	1.09 [0.92–1.29](p = 0.318)	0.95 [0.76–1.20](p = 0.671)
**Neutrophils (×10^9^ cells/L)**	**1.06 [0.86–1.30](p = 0.598)**	1.04 [0.84–1.28](p = 0.738)	0.96 [0.87–1.05](p = 0.355)	1.10 [0.93–1.31](p = 0.282)	1.16 [0.57–1.43](p = 0.150)	0.84 [0.57–1.23](p = 0.371)
**Monocytes (×10^9^ cells/L)**	**0.65 [0.03–16.7](p = 0.794)**	6.69 [0.27–166.4](p = 0.246)	1.15 [0.51–2.61](p = 0.739)	0.89 [0.13–6.21](p = 0.903)	0.26 [0.03–2.69](p = 0.257)	1.92 [0.32–11.4](p = 0.472)
**Lymphocytes (×10^9^ cells/L)**	0.28 [0.05–1.79](p = 0.179)	4.59 [0.93–22.6](p = 0.061)	1.48 [0.91–2.40](p = 0.113)	0.67 [0.29–1.58](p = 0.359)	0.58 [0.23–1.45](p = 0.241)	2.76 [0.87–8.77](p = 0.085)
**NLR**	1.12 [1.01–1.25](p = 0.041)	0.92 [0.79–1.07](p = 0.295)	0.89 [0.78–1.01](p = 0.059)	1.04 [0.91–1.19](p = 0.536)	**1.27 [1.01–1.60](p = 0.043)**	0.61 [0.30–1.24](p = 0.172)
**MLR transformed**	68.6 [3.36–1,403](p = 0.006)	0.13 [0.01–2.18](p = 0.158)	0.28 [0.05–1.67](p = 0.162)	1.37 [0.16–11.8](p = 0.777)	2.32 [0.24–22.4](p = 0.467)	0.267 [0.00–31.9](p = 0.584)

Odds ratios, hazard ratios, 95% confidence intervals, and their respective p-values are shown for all correlations. Significant values (p < 0.05) are highlighted in bold. ICU, Intensive Care Unit; MLR, Monocyte-to-Lymphocyte Ratio; mRS, modified Rankin Scale; NLR, Neutrophil-to-Lymphocyte Ratio; WCC, White Cell Count.

Accounting for patients with inadequate blood samples (12 patients) and those lost to follow-up (an additional 4 patients), the final multivariable regression model was performed in 45 patients for in-hospital outcomes (treatment failure and ICU admission) and 41 patients for long-term outcomes (mRS, mortality, and relapse). The multivariable regression analysis showed that age was associated with worse 12-month functional outcomes, but not long-term mRS (**Tables 5** and **6**). First line treatment failure corresponded with increased odds of ICU admission. Higher NLR had a significant relationship with failure of first line immunotherapy (OR 1.32, 95% CI 1.01–1.74, p = 0.044). Transformed MLR, NMDAR serostatus, sex, the presence of tumor, and delay in treatment were not related to any of our outcomes.

**Table 5:** Multivariable logistic regression analysis for NLR for the whole cohort.

	Mortality	mRS ≤2 at 12-months	mRS ≤2 at final	ICU admission	Failure of first line treatment	Relapse
NMDAR serostatus (Ref: positive)	–	7.93 [0.25–249.2](p = 0.239)	0.84 [0.22–3.16](p = 0.972)	0.32 [0.02–5.04](p = 0.421)	1.27 [0.12–13.6](p = 0.842)	–
Age	1.08 [0.96–1.22](p = 0.183)	**0.92 [0.84–0.99](p = 0.044)**	1.02 [0.98–1.05](p = 0.311)	0.98 [0.92–1.05](p = 0.610)	0.99 [0.93–1.04](p = 0.575)	1.06 [0.98–1.15](p = 0.164)
Sex (Ref: female)	82.6 [0.50–13595](p = 0.090)	1.20 [0.12–11.9](p = 0.875)	0.86 [0.29–2.62](p = 0.796)	0.16 [0.02–1.29](p = 0.085)	0.53 [0.08–3.42](p = 0.506)	1.38 [0.07–28.0](p = 0.834)
Tumor	188.2 [0.15–237568](p = 0.151)	–	1.57 [0.48–5.10](p = 0.454)	–	–	–
Treatment delay (days)	0.97 [0.93–1.01](p = 0.184)	–	1.00 [0.99–1.00](p = 0.999)	–	1.00 [0.99–1.01](p = 0.448)	1.00 [0.99–1.01](p = 0.815)
First line treatment failure	–	–	–	**11.4 [1.52–86.2](p = 0.018)**	–	–
NLR	1.54 [0.99–2.38](p = 0.055)	0.91 [0.76–1.09](p = 0.316)	0.87 [0.75–1.00](p = 0.053)	0.96 [0.81–1.13](p = 0.596)	**1.32 [1.01–1.74](p = 0.044)**	0.74 [0.33–1.66](p = 0.470)

Odds ratios, hazard ratios, 95% confidence intervals, and their respective p-values are shown for all correlations. Significant values (p < 0.05) are highlighted in bold. ICU, Intensive Care Unit; MLR, Monocyte-to-Lymphocyte Ratio; mRS, modified Rankin Scale; NLR, Neutrophil-to-Lymphocyte Ratio; WCC, White Cell Count.

**Table 6:** Multivariable logistic regression analysis for MLR transformed for the whole cohort.

	Mortality	mRS ≤2 at 12-months	mRS ≤2 at final	ICU admission	Failure of first line treatment	Relapse
NMDAR serostatus (Ref: positive)	–	10.1 [0.30–339.5](p = 0.199)	0.76 [0.21–2.80](p = 0.680)	0.30 [0.02–4.71](p = 0.394)	1.38 [0.17–11.2](p = 0.764)	–
Age	1.03 [0.94–1.12](p = 0.593)	**0.91 [0.83–0.99](p = 0.038)**	1.02 [0.99–1.05](p = 0.248)	0.98 [0.92–1.05](p = 0.605)	0.98 [0.94–1.03](p = 0.519)	1.07 [0.99–1.16](p = 0.089)
Sex (Ref: female)	15.5 [0.69–349.1](p = 0.084)	1.48 [0.14–15.2](p = 0.741)	0.92 [0.34–2.50](p = 0.865)	0.17 [0.02–1.40](p = 0.100)	0.65 [0.14–3.06](p = 0.585)	7341 [0.13–24.0](p = 0.682)
Tumor	10.6 [0.16–703.4](p = 0.269)	–	1.42 [0.45–4.51](p = 0.555)	–	–	–
Treatment delay (days)	0.99 [0.96–1.02](p = 0.356)	–	1.00 [1.00–1.01](p = 0.948)	–	1.00 [0.99–1.01](p = 0.792)	1.00 [0.99–1.01](p = 0.882)
First line treatment failure	–	–	–	**9.52 [1.47–61.6](p = 0.018)**	–	–
MLR transformed	4.45 [0.76–25.5](p = 0.094)	0.37 [0.06–2.13](p = 0.265)	0.56 [0.27–1.14](p = 0.110)	1.00 [0.30–3.40](p = 0.998)	1.22 [0.46–3.24](p = 0.687)	0.24 [0.01–4.13](p = 0.329)

Odds ratios, hazard ratios, 95% confidence intervals, and their respective p-values are shown for all correlations. Significant values (p < 0.05) are highlighted in bold. ICU, Intensive Care Unit; MLR, Monocyte-to-Lymphocyte Ratio; mRS, modified Rankin Scale; NLR, Neutrophil-to-Lymphocyte Ratio; WCC, White Cell Count.

## Discussion

Our study examines the role of peripheral blood immune cell counts and ratios as a prognostic biomarker in AE. High NLR at the time of hospital admission may predict an increased chance of first line treatment failure. MLR was not associated with any outcome.

The NLR was first introduced to AE research in 2019 (23). It was found that 34 cases of AE with neuronal cell surface autoantibodies had a significantly higher median NLR compared to 35 healthy controls. In this study, higher NLR was associated with higher mRS at the time of admission both in the whole AE cohort and the anti-NMDAR subset. The optimal cut-off to predict severe disability (mRS >2) was an NLR >4.82 (AUC 0.875). A subsequent study examined a group of 50 AE cases and their long-term functional outcomes (10). The investigators found that after a median of 11 months follow-up, the NLR on admission was associated with higher odds of mRS >1 (OR 2.17, 95% CI 1.03–4.57). Interestingly, more than 60% of the study participants reached mRS ≤1 while only 40% of the cohort were treated with immunotherapy. In this cohort, the vast majority of the participants had no detectable diagnostic antibody (82%), and half the total cohort only met criteria for possible rather than definite AE. The optimal NLR value to predict mRS >1 was identified as 4.45 (AUC 0.866). A third retrospective study of 29 patients with anti-NMDAR encephalitis found that the median NLR was significantly higher in non-responders to first line immunotherapy than in responders, both before (4.10 *vs* 2.39) and after (4.14 *vs* 2.62) treatment (11). The results from our study support the above observations, with 1.3-fold increase in odds of treatment failure for every rise in the NLR by 1 unit. Our study did not find a relationship between NLR and mRS as was reported in a previous study (10). This discrepancy could be due to differences in the study cohort or outcome definitions as described above. In our study, we explored a potential novel immunological prognostic biomarker, MLR. The transformed (normalized) MLR had no relationship with clinical outcomes.

While AE with neuronal cell surface antibodies is currently considered to be largely mediated by adaptive immunity, the exact underlying immunopathological mechanisms behind immune activation and propagation are currently not fully understood. Similarly, MS was traditionally considered to be a disease of adaptive immunity, however there are several robust and large studies that find NLR and MLR to be biomarkers of long-term prognosis in MS. NLR has also been shown to be a biomarker for disease activity in many other conditions, including systemic lupus erythematosus (5), ulcerative colitis (2), and rheumatoid arthritis (4). A detailed review describes potential roles for innate immune cells, cytokines, and chemokines in AE (1), and highlights some of the gaps in understanding this area. Hence, we believe NLR would serve as a potential biomarker in AE through a novel concept, requiring further investigation. The exact pathophysiological mechanism that underpins the relationship between NLR and first line treatment failure in this study is unclear. Neutrophils are an essential part of the early inflammatory cascade, and contribute to breakdown of the blood-brain barrier (24, 25). We hypothesize that dysregulated innate immune activation, increased circulating neutrophils and sequestration of lymphocytes are indicative of a heightened inflammatory response. Hence NLR may be representative of disease activity, as suggested in a prior study (23).

As expected, older age was associated with poorer 12-month functional outcomes however was not predictive of mortality, likely because death was uncommon. It was also interesting to note that time to immunotherapy, a well-established marker of poor prognosis, was not related to any of any of the outcomes in our cohort. It is possible that this reflects evolving clinical practice over time compared to previous studies. Early recognition and treatment of AE, including prompt escalation to second line immunotherapy, are now commonplace and long treatment delays are therefore very uncommon. We found a significant relationship between ICU admission and first-line treatment failure. It is important to highlight that most of these patients were already admitted to intensive care when their treatment failed. This implies that critical illness was an inevitable part of the disease history and not a result of poor responsiveness to immunotherapy.

The use of the mRS to assess neurological outcome after AE is a limitation. This scale was originally developed and validated to assess neurological outcomes after stroke, and is therefore heavily weighted towards physical disability (18). In lieu of another tool to examine neurological outcomes in AE, the literature has largely adopted the mRS to describe long-term sequelae (26). One of the key advantages of the mRS is that it is straightforward and can be applied retrospectively. However, mRS does not reflect some of the neurological sequelae of AE, such as drug-resistant epilepsy, mood disorders, or cognitive impairment. As a result, mRS may be insensitive to meaningful improvements in neurological status after AE. Tools that might be better suited to demonstrate disease-specific outcomes, such as cognitive scores, quality of life or productivity measure were not available in this retrospective study. This demonstrates the need for on prospectively designed observational studies which include more appropriate and disease-tailored outcome measures. One example is the Clinical Assessment Scale in Autoimmune Encephalitis (CASE) (27), which evaluates many domains affected by AE. It was found to have strong inter-observer reliability and is also correlated with mRS. Ideally, comprehensive evaluation of patients with AE should include assessment of cognitive function, productivity, and quality of life. Retrospective design and limited case numbers are other limitations of this study.

Our study was also limited to cases with neuronal cell surface antibodies, where neuronal dysfunction is thought to be largely antibody-mediated (28). It is possible that studies in NLR and MLR could be more informative in cases with intracellular autoantibodies, which are thought to be predominantly T-cell mediated immune response and are characterized pathologically by high numbers of infiltrating macrophages and microglial cells (28).

## Conclusion

Our data indicates that the NLR represents a valuable biomarker that contributes to the prognostic information with regards to response to first-line therapy. This information has important clinical implications; possibly suggesting that physicians should have a lower threshold for initiation of second-line immunotherapy in patients with a high NLR. This may give rise to future treatment guidelines but warrants further investigation. MLR was not seen to have a relationship with any outcome in this small study.

## Data Availability Statement

The original contributions presented in the study are included in the article/supplementary materials. Further inquiries can be directed to the corresponding author.

## Ethics Statement

The studies involving human participants were reviewed and approved by Melbourne Health Human Research Ethics Committee. Written informed consent for participation was not required for this study in accordance with the national legislation and the institutional requirements.

## Author Contributions

JB: Study concept and design, data collection, data interpretation and statistics, drafted the manuscript. RW: Data collection, revised the manuscript for intellectual content. US: Revised the manuscript for intellectual content. CK: Data collection, revised the manuscript for intellectual content. PB: Data collection, revised the manuscript for intellectual content. KB: Data collection, revised the manuscript for intellectual content. CN: Data collection, revised the manuscript for intellectual content. WD’S: Revised the manuscript for intellectual content. AB: Data collection, revised the manuscript for intellectual content. TK: Revised the manuscript for intellectual content, revised statistical design. HB: Revised the manuscript for intellectual content. TO’B: Study concept and design, revised the manuscript for intellectual content. MM: Study concept and design, data interpretation and statistics, revised the manuscript for intellectual content. All authors contributed to the article and approved the submitted version.

## Conflict of Interest

WD’S’s salary is part-funded by The University of Melbourne. He has received travel, investigator-initiated, scientific advisory board and speaker honoraria from UCB Pharma Australia & Global; investigator-initiated, scientific advisory board, travel and speaker honoraria from Eisai Australia & Global; advisory board honoraria from Liva Nova; educational grants from Novartis Pharmaceuticals, Pfizer Pharmaceuticals and Sanofi-Synthelabo; educational, travel and fellowship grants from GSK Neurology Australia, and honoraria from SciGen Pharmaceuticals. AB is on the editorial boards for *Neurology* and *International Stroke Journal*, and an Australian Scientific Advisory Board for Biogen. TK served on scientific advisory boards for Roche, Celgene, Sanofi-Genzyme, Novartis, Merck, and Biogen, steering committee for Brain Atrophy Initiative by Sanofi-Genzyme, received conference travel support and/or speaker honoraria from WebMD Global, Novartis, Biogen, Sanofi-Genzyme, Teva, BioCSL, and Merck and received research support from Biogen. HB’s institution receives funding from Biogen, Roche, Merck, and Novartis for speaker engagements, study steering and advisory committee service. He is on the editorial board of *Multiple Sclerosis and Related Disorders* and the Steering committee of the Brain Health Initiative (Oxford Health Policy Forum). TO’B receives research funding from Biogen, UCB Pharma, Eisai Pharma, Anavex Pharmaceuticals, Zynerba Pharmaceuticals, and serves on the scientific advisory boards for UCB Pharma, Eisai Pharmaceuticals, Zynerba Pharmaceuticals, ES Therapeutics, Seqirus Pharmaceuticals. MM’s institution receives funding from Merck.

The remaining authors declare that the research was conducted in the absence of any commercial or financial relationships that could be construed as a potential conflict of interest.

## Appendix Australian Autoimmune Encephalitis Consortium list of co-investigators

**Table:**

Name	Location
Sarah Griffiths	Monash University, Melbourne, Vic, Australia
Tracie Tan	Monash University and Melbourne Health, Vic, Australia
Charles Malpas	The University of Melbourne, Melbourne Health, Melbourne, Vic, Australia
Rubina Alpitsis	Alfred Health, Melbourne, Vic, Australia
Richard Macdonell	Austin Health, Melbourne, Vic, Australia
David Tarlinton	Department of Immunology, Monash University, Melbourne, Vic, Australia
Steve Reddel	Sydney Brain and Mind, University of Sydney, NSW, Australia
Todd Hardy	Concord Hospital, NSW, Australia
Bruce Taylor	Royal Hobart Hospital, Hobart, Australia
Brian Long	Monash Medical Centre, Vic, Australia
Tissa Wijeratne	Western Health, Vic, Australia
Owen White	Monash University and Alfred Health, Melbourne, Vic, Australia
Jo Fielding	Monash University, Melbourne, Vic, Australia
Matt Ligtermoet	Northern Health, Melbourne, Victoria, Australia
Meng Tan	Gold Coast Health, UQ, Queensland, Australia
Jayashri Kulkarni	Alfred Health and Monash University, Melbourne, Vic, Australia
Robert Bourke	Melbourne Health, Vic, Australia
Dennis Velakoulis	The University of Melbourne, Melbourne Health, Melbourne, Vic, Australia
Ernie Butler	Peninsula Health, Melbourne, Vic, Australia
Meaghan Clough	Monash University, Melbourne, Vic, Australia
